# Genetic and epigenetic alterations of netrin-1 receptors in gastric cancer with chromosomal instability

**DOI:** 10.1186/s13148-015-0096-y

**Published:** 2015-07-23

**Authors:** Keisuke Toda, Takeshi Nagasaka, Yuzo Umeda, Takehiro Tanaka, Takashi Kawai, Tomokazu Fuji, Fumitaka Taniguchi, Kazuya Yasui, Nobuhito Kubota, Yuko Takehara, Hiroshi Tazawa, Shunsuke Kagawa, Dong-Sheng Sun, Naoshi Nishida, Ajay Goel, Toshiyoshi Fujiwara

**Affiliations:** Department of Gastroenterological Surgery, Okayama University Graduate School of Medicine, Dentistry and Pharmaceutical Sciences, Okayama City, Okayama 700-8558 Japan; Department of Pathology, Okayama University Graduate School of Medicine, Dentistry and Pharmaceutical Sciences, Okayama City, Okayama 700-8558 Japan; Department of Oncology, Kailuan General Hospital in Tangshan of Hebei Province, Tangshan, Hebei 063000 China; Department of Gastroenterology and Hepatology, Kinki University Faculty of Medicine, 337-2 Ohno-higashi, Osaka-sayama, Osaka 589-8511 Japan; Center for Gastrointestinal Cancer Research, Center for Epigenetics, Cancer Prevention and Cancer Genomics, Baylor Research Institute and Charles A Sammons Cancer Center, Baylor University Medical Center, Dallas, TX 75246 USA

**Keywords:** Gastric cancer, Methylation, Chromosomal instability, *DCC*, *UNC5C*, Netrin-1 receptors

## Abstract

**Background:**

The gene expressions of netrin-1 dependence receptors, *DCC* and *UNC5C*, are frequently downregulated in many cancers. We hypothesized that downregulation of DCC and UNC5C has an important growth regulatory function in gastric tumorigenesis.

**Results:**

In the present study, a series of genetic and epigenetic analyses for *DCC* and *UNC5C* were performed in a Japanese cohort of 98 sporadic gastric cancers and corresponding normal gastric mucosa specimens. Loss of heterozygosity (LOH) analyses and microsatellite instability (MSI) analysis was applied to determine chromosomal instability (CIN) and MSI phenotypes, respectively. More than 5 % methylation in the *DCC* and *UNC5C* promoters were found in 45 % (44/98) and 32 % (31/98) gastric cancers, respectively, and in 9 % (9/105) and 5 % (5/105) normal gastric mucosa, respectively. Overall, 70 % (58 of 83 informative cases) and 51 % (40 of 79 informative cases) of gastric cancers harbored either LOH or aberrant methylation in the *DCC* and *UNC5C* genes, respectively. In total, 77 % (51 of 66 informative cases) of gastric cancers showed cumulative defects in these two dependence receptors and were significantly associated with chromosomal instability. Both DCC and UNC5C were inactivated in 97 % of CIN-positive gastric cancers and in 55 % of CIN-negative gastric cancers.

**Conclusions:**

Defect in netrin receptors is a common feature in gastric cancers. *DCC* alterations are apparent in the early stages, and *UNC5C* alterations escalate with the progression of the disease, suggesting that the cumulative alterations of netrin-1 receptors was a late event in gastric cancer progression and emphasizing the importance of this growth regulatory pathway in gastric carcinogenesis.

**Electronic supplementary material:**

The online version of this article (doi:10.1186/s13148-015-0096-y) contains supplementary material, which is available to authorized users.

## Background

Global estimates of cancer incidence rank gastric cancer as the fourth most common malignancy and the second most common cause of cancer-related deaths worldwide [1]. Gastric cancer is a heterogeneous disease with multiple environmental etiologies and with alternative pathways of carcinogenesis [2, 3]. One of the major etiological risk factors for gastric cancer is *Helicobacter pylori* (*H. pylori*) infection. Previous reports indicated a 93.1–100 % infection rate for *H. pylori* in patients with gastric cancer, while only 1.2–2.8 % of individuals infected with *H. pylori* develop gastric cancer [4–7].

Current knowledge on the molecular mechanisms underlying gastric carcinogenesis indicates one major epigenetic instability pathway and two major genetic instability pathways [8]. The major epigenetic instability pathway is defined as the CpG island methylator phenotype (CIMP), which was initially described in colorectal cancer and also observed in a subset of gastric cancers and which harbors a critical degree of aberrant promoter hypermethylation associated with transcriptional silencing of multiple tumor suppressor genes [9, 10]. The two major genetic instability pathways include microsatellite instability (MSI) and chromosomal instability (CIN) [8]. MSI is defined as the presence of replication errors in simple repetitive microsatellite sequences caused by mismatch repair (MMR) deficiencies. One is Lynch syndrome caused by germline mutations in MMR genes and another is sporadic MSI caused mainly by promoter hypermethylation in the *MLH1* gene [10, 11]. On the other hand, CIN, which is characterized by chromosomal alterations—either qualitative or quantitative—is a more common pathway that may comprise clinicopathologically and molecularly heterogeneous tumors [8].

The Cancer Genome Atlas Research Network recently divided gastric cancers into four subtypes [12]. Tumors were first categorized by Epstein–Barr virus (EBV)-positivity (9 %), then by MSI-high status, hereafter called MSI-positive (22 %), and the remaining tumors were classified by degree of aneuploidy into those termed genomically stable (20 %) or those exhibiting CIN (50 %). EBV-positive cancers as well as MSI-positive cancers were known to cluster each on its own, exhibiting extreme CIMP. Differences between the EBV-CIMP and MSI-associated gastric-CIMP methylation profiles are exemplified by the fact that all EBV-positive tumors assayed displayed *CDKN2A* (*p16INK4A*) promoter hypermethylation but lacked the *MLH1* hypermethylation characteristic of MSI-associated CIMP.

With respect to CIN characterized by copy number changes in chromosomes, Deng et al. used high resolution genomic analysis to profile somatic copy number alterations in a panel of 233 gastric cancers (primary tumors and cell lines) and 98 matched gastric non-malignant tissues. Regarding broad chromosomal regions, the most frequently amplified region included chromosomes 1q, 5p, 6p, 7p, 7q, 8q, 13q, 19p, 20p, and 20q, and the most frequently deleted regions included chromosomes 3p, 4p, 4q, 5q, 6q, 9p, 14q, 18q, and 21q [13].

Frequently deleted chromosomal regions are usually characterized by loss of heterozygosity (LOH) and suggest the presence of tumor suppressor genes [14, 15]. LOH on chromosome 18q21 is found in 30–71 % of gastric cancers [13, 16–18], and *DPC4* (*Smad4*)/*DCC* have been postulated to be the major targets. *DPC4* (*Smad4*), a tumor suppressor gene, exhibits frequent mutations accompanied by LOH in approximately 20 % of pancreatic cancers [19], but no mutations have been reported in gastric cancers [20]. In contrast, few studies have focused on *DCC* gene alterations, and its genetic/epigenetic status still remains virtually unexplored in gastric cancer, partly because of the length and complexity of this gene [21]. Interestingly, recent studies have demonstrated that DCC as well as UNC5C serve as dependence receptors for netrin-1, thus, reinforcing their potential role as tumor-suppressors in human cancers [22–25].

DCC receptors are distributed along the length of the epithelium in the intestine, whereas netrin-1 is differentially expressed, forming a gradient within the gastrointestinal tract [24]. A high concentration of netrin-1 is present at the crypt base where stem cells and transient amplifying cells reside. By contrast, a low concentration of netrin-1 exists at the tip of the villi, where many cells are undergoing apoptosis and sloughing-off. This netrin-1 gradient was examined further using transgenic mice to determine if netrin-1 is responsible for regulating DCC-induced apoptosis in the intestinal epithelium [24]. The study by Mazelin et al. indicated that netrin-1 overexpression caused a decrease in intestinal epithelial cell death, whereas no increase in proliferation and differentiation of cells was observed. By contrast, netrin-1–mutant newborn mice exhibited increased cell death. Taken together, these data support the concept that netrin-1 regulates apoptosis through the DCC-dependence receptor in the intestine. However, netrin-1 is unlikely to be a direct regulator of intestinal homeostasis, given that normal epithelial organization is not disrupted by netrin-1 overexpression [24].

Similar to DCC receptors, other netrin-1 receptors, including UNC5A, UNC5B, and UNC5C, were also discovered as putative tumor suppressor genes in various tumors, including gastric cancer [26, 27]. In particular, a twofold downregulation of UNC5C expression compared with the corresponding normal tissues was observed in approximately 70 % of gastric cancer cases [26]. This region is located at 4q21–23, which is often a site of deletion in gastric cancer and is associated with epigenetic gene inactivation, such as promoter methylation [26–28].

In this study, we hypothesized that downregulation of DCC and UNC5C plays an important growth regulatory function in gastric tumorigenesis, which we addressed by investigating a panel of gastric cancer cell lines and clinical specimens from patients with gastric cancer. Herein, we report that the majority of gastric cancers show loss of both netrin-1 receptors. We also provide data suggesting that the inactivation of these receptors is mediated through both genetic and epigenetic mechanisms. Cumulative defects in these two dependence receptors are significantly associated with the CIN phenotype, emphasizing the importance of these novel findings and this growth regulatory pathway in gastric carcinogenesis.

## Results

### Characteristics of gastric cancer patients

Of 98 gastric cancer patients, 34 patients were female (35 %), and 48 tumors were pathologically diagnosed as differentiated (49 %) (Table 1). With regard to TNM stage, 18, 29, 37, and 14 gastric cancer patients were classified as stage I, II, III, and IV, respectively. By tumor genetic analyses, 13 gastric cancers were categorized as displaying microsatellite instability (MSI; 13 %). The mean LOH ratio of the 98 tumors was 0.24 (standard deviation (SD), ±0.3).

**Table 1:** Characteristics of gastric cancer patients

Characteristic		Percentage (No.)
Age	Mean age (SD)	65.1 (11.8)
Gender	Female	35 (34)
	Male	65 (64)
Histology	Diff	49 (48)
	Undiff	51 (50)
Stage	IA/IB	18 (18)
	IIA/IIB	30 (29)
	IIIA/IIIB/IIIC	38 (37)
	IV	14 (14)
T	T1a/1b	14 (14)
	T2	14 (14)
	T3	28 (27)
	T4a/4b	44 (43)
N	N0	27 (26)
	N1	35 (34)
	N2	24 (24)
	N3	14 (14)
Distant metastasis	Negative	86 (84)
	Positive	14 (14)
MSI	MSI	13 (13)
	Non-MSI	87 (85)
*LOH Ratio*	Mean Ratio (SD)	0.24 (0.3)
CIN	Positive	51 (50)
	Negative	47 (46)
	Not informative	2 (2)
*KRAS*	Mutant	5 (5)
	Wild	95 (93)
*BRAF*	Mutant	0 (0)
	Wild	100 (98)
*PIK3CA*	Mutant	4 (3)
	Wild	96 (94)
H.pyroli	Positive	71 (70)
	Negative	29 (28)

CIN phenotype was categorized by calculating the LOH ratio of the informative markers of the seven polymorphic microsatellite sequences, independently from the 4q and 18q loci. When a tumor showed a LOH ratio higher than 0, the tumor was categorized as CIN-positive. By this criterion, 50/98 tumors (51 %) were classified as CIN-positive.

Direct sequencing of gastric cancer specimens revealed the proportion of *KRAS*, *BRAF*, and *PIK3CA* mutations (Table 1). Mutations were detected in the *KRAS* codon 12 (5 %, *N* = 5/98) and codon 13 (1 %, *N* = 1/98); *BRAF* codon 600 (0 %, *N* = 0/98); *PIK3CA* codon 545 (1 %, *N* = 1/98); and codon 1047 (3 %, *N* = 3/98). *KRAS* codon 12 mutations consisted of G12D (35G to A, *N* = 4) and G12R (34G to C, *N* = 1), and codon 13 mutations included G13D (38G to A, *N* = 1). Interestingly, one tumor displayed both *KRAS* codon 12 and 13 mutations (Additional file 1: Figure S1A). *PIK3CA* exon 9 mutations comprised E545K (1633G to A, *N* = 1), while exon 20 mutations comprised H1047R (3140A to G, *N* = 3). Furthermore, we determined the infection status of *H. pylori* by recovering the *cagA* genotype (Additional file 1: Figure S1B). Through this analysis, we could recover the *cagA* sequence from 70 gastric cancer tissues (71 %).

### Methylation status of *DCC* in gastric cancer specimens and association with clinicopathological features

**Fig. 1:**
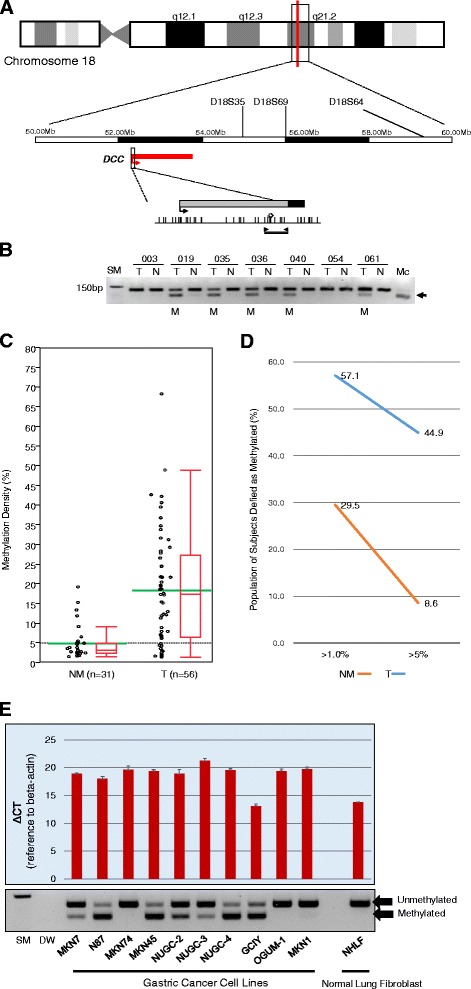
*DCC* promoter methylation and 18q LOH analyses. (**a**) Schematic representation of the location of the three LOH probes and *DCC* gene promoter regions in chromosome 18. The *red line* denotes the *DCC* gene. *Gray* and *black squares* represent the untranslated and the coding exon 1 regions, respectively; *arrows on the squares* indicate transcriptional starting sites; *vertical lines* indicate CpG sites; *white diamonds* represent the restriction sites for HhaI; *thick horizontal lines* depict the locations of COBRA products; *arrows on the thick horizontal lines* denote COBRA primers. (**b**) Representative results of COBRA of *DCC. Arrows* indicate methylated alleles; *M* denotes methylation; *U* denotes unmethylation; *Mc* denotes the methylated control; *SM* denotes the size marker. (**c**) Results of *DCC* methylation as a continuous variable. In the box plot diagrams, the *horizontal line* within each box represents the median, the *limits* of each box represent the interquartile ranges, and the *whiskers* are the maximum and minimum values. Each *green bar* represents the mean. *NM* denotes normal mucosa. *T* denotes tumor. (**d**) The frequency of methylation-positivity of cancer and normal tissues according to different thresholds. (**e**) *DCC* mRNA expression levels and methylation status in 10 gastric cancer cell lines and a human lung fibroblast cell line. *DCC* mRNA expression is observed (lower ΔC_T_) in GCIY and NHLH cell lines. DW denotes distilled water

We investigated *DCC* methylation status in 98 gastric cancers and 105 normal gastric mucosa specimens. Location of the *DCC* gene and the results of a panel of representative combined bisulfite restriction analyses (COBRA) are depicted in Fig. 1a–b; these results were analyzed as continuous variables (Fig. 1c). We found that 56/98 gastric cancers (57 %) and 31/105 normal gastric mucosa specimens (29.5 %) displayed more than 1.0 % methylation in the *DCC* promoter. The mean methylation level was 18.3 % [95 % confidence interval (CI), 14.5–22.2 %] among gastric cancer tissues that displayed over 1.0 % methylation in the *DCC* promoter and 4.9 % (95 % CI, 3.3–6.5 %) in the corresponding normal gastric mucosa specimens that displayed over 1.0 % methylation (*P* < 0.0001, Wilcoxon/Kruskal–Wallis test; Fig. 1c–d). Therefore, we defined a *DCC* methylation of 5 % or more as a continuous variable (i.e., >5.0 % methylation was defined as methylation-positive (methylated) and <5.0 % methylation as methylation-negative (unmethylated)). Using this criterion, we observed *DCC* methylated cases in 44/98 gastric cancers (45 %) and in 9/105 normal gastric mucosa (9 %).

Next, we investigated the association between *DCC* promoter methylation and various clinicopathological and genetic features. *DCC* methylation status was significantly associated with MSI status. MSI-positive gastric cancers were significantly more frequently associated with *DCC* methylation than with *DCC* unmethylation (23 vs. 6 %, *P* = 0.013; Table 2). There were no significant associations between *DCC* methylation status and any other variables.

**Table 2:** Association between epigenetic/genetic alterations of the DCC gene and clinicopathological features in gastric cancers

		*DCC* Methylation Status—% (No.)			18q LOH Status—% (No.)				*DCC* Alteration Status—% (No.)						
		Unmethylation	Methylation	*P*	Not informative	Negative	Positive	*P*	Not informative	Negative	Positive	Positive			*P*
											Total	Methylation alone	LOH alone	Both	
		(*n* = 54)	(*n* = 44)		(*n* = 15)	(*n* = 47)	(*n* = 36)		(*n* = 15)	(*n* = 25)	(*n* = 58)	(*n* = 22)	(*n* = 19)	(*n* = 17)	
Age	Mean age (SD)	63.6 (12.5)	67.0 (10.7)	0.48^a^	68.9 (11.4)	64.8 (11.4)	63.9 (12.4)	0.76^a^	68.9 (11.4)	63.5 (12.9)	64.8 (11.3)	66.3 (9.5)	60.7 (12.7)	67.4 (11.4)	0.98^a^
Gender	Female	39 (21)	30 (13)	0.33^b^	40 (6)	38 (18)	28 (10)	0.32^b^	40 (6)	44 (11)	29 (17)	32 (7)	32 (6)	24 (4)	0.19^b^
	Male	61 (23)	70 (31)		60 (9)	62 (29)	72 (26)		60 (9)	56 (14)	71 (41)	68 (15)	68 (13)	76 (13)	
Histology	Diff	41 (22)	59 (26)	0.071^b^	47 (7)	55 (26)	42 (15)	0.22^b^	47 (7)	52 (13)	48 (28)	59 (13)	32 (6)	53 (9)	076^b^
	Undiff	59 (32)	41 (18)		53 (8)	45 (21)	58 (21)		53 (8)	48 (12)	52 (30)	41 (9)	68 (13)	47 (8)	
Stage	IA/IB	20 (11)	16 (7)	0.83^b^	13 (2)	19 (9)	19 (7)	0.15^b^	13 (2)	24 (6)	17 (10)	14 (3)	21 (4)	18 (3)	0.70^b^
	IIA/IIB	26 (14)	34 (15)		27 (4)	30 (14)	31 (11)		27 (4)	24 (6)	33 (19)	36 (8)	32 (6)	29 (5)	
	IIIA/IIIB/IIC	39 (21)	36 (16)		53 (8)	43 (20)	25 (9)		53 (8)	40 (10)	33 (19)	45 (10)	26 (5)	24 (4)	
	IV	15 (8)	14 (6)		7 (1)	9 (4)	25 (9)		7 (1)	12 (3)	17 (10)	5 (1)	21 (4)	29 (5)	
T	T1a/1b	15 (8)	14 (6)	0.24^b^	7 (1)	17 (8)	14 (5)	0.94^b^	7 (1)	16 (4)	16 (9)	18 (4)	21 (4)	6 (1)	0.54^b^
	T2	13 (7)	16 (7)		13 (2)	15 (7)	14 (5)		13 (2)	16 (4)	14 (8)	14 (3)	11 (2)	18 (3)	
	T3	20 (11)	36 (16)		33 (5)	28 (13)	25 (9)		33 (5)	16 (4)	31 (18)	41 (9)	16 (3)	35 (6)	
	T4a/b	52 (28)	34 (15)		47 (7)	40 (19)	47 (17)		47 (7)	52 (13)	40 (23)	27 (6)	53 (10)	41 (7)	
N	N0	30 (16)	23 (10)	0.66^b^	20 (3)	28 (13)	28 (10)	0.29^b^	20 (3)	32 (8)	26 (15)	23 (5)	32 (6)	24 (4)	0.19^b^
	N1	37 (20)	32 (14)		47 (7)	38 (18)	25 (9)		47 (7)	44 (11)	28 (16)	32 (7)	26 (5)	24 (4)	
	N2	20 (11)	30 (13)		20 (3)	26 (12)	25 (9)		20 (3)	20 (5)	28 (16)	32 (7)	21 (4)	29 (5)	
	N3	13 (7)	16 (7)		13 (2)	9 (4)	22 (8)		13 (2)	4 (1)	19 (11)	14 (3)	21 (4)	24 (4)	
Distant metastasis	Negative	85 (46)	86 (38)	0.87^b^	93 (14)	91 (43)	75 (27)	0.041^b^	93 (14)	88 (22)	83 (48)	95 (21)	79 (15)	71 (12)	0.55^b^
	Positive	15 (8)	14 (6)		7 (1)	9 (4)	25 (9)		7 (1)	12 (3)	17 (10)	5 (1)	21 (4)	29 (5)	
MSI status	MSI	6 (3)	23 (10)	0.013^b^	33 (5)	9 (4)	11 (4)	0.69^b^	33 (5)	4 (1)	12 (7)	14 (3)	0 (0)	24 (4)	0.25^b^
	Non-MSI	94 (51)	77 (34)		67 (10)	91 (43)	89 (32)		67 (10)	96 (24)	88 (51)	86 (19)	100 (19)	76 (13)	
LOH ratio	Mean ratio (SD)	0.23 (0.30)	0.26 (0.31)	0.55^a^	0.22 (0.29)	0.10 (0.15)	0.44 (0.36)	<0.0001^a^	0.22 (0.29)	0.05 (0.10)	0.33 (0.33)	0.16 (0.18)	0.44 (0.34)	0.44 (0.39)	<0.0001^a^
CIN*	Positive	46 (25)	57 (25)	0.39^b^	47 (7)	38 (18)	74 (25)	0.0017^b^	47 (7)	24 (6)	66 (37)	55 (12)	76 (13)	71 (12)	0.0005^b^
	Negative	54 (27)	43 (19)		53 (8)	62 (29)	26 (9)		53 (8)	76 (19)	34 (19)	45 (10)	24 (4)	29 (5)	
*KRAS*/*BRAF*/*PIK3CA*	Mutant	6 (3)	11 (5)	0.30^b^	27 (4)	6 (3)	3 (1)	0.45^b^	27 (4)	4 (1)	5 (3)	9 (2)	5 (1)	0 (0)	0.82^b^
	Wild	94 (51)	89 (38)		73 (11)	94 (44)	97 (35)		73 (11)	96 (24)	95 (55)	91 (20)	95 (18)	100 (17)	
H.pyroli	Positive	72 (39)	70 (31)	0.85^b^	80 (12)	66 (31)	75(27)	0.37^b^	80 (12)	60 (15)	74 (43)	73 (16)	79 (15)	71 (12)	0.20^b^
	Negative	28 (15)	30 (13)		20 (3)	34(16)	25 (9)		20 (3)	40 (10)	26 (15)	27 (6)	21 (4)	29 (5)	

*Two cases are not informative of CIN status

^a^ *P* value were calculated between unmethylation and methylation, 18qLOH negative and positive, and DCC alteration negative and positive(total) by Wilcoxon/Kruskal–Wallis test

^b^ *P* values were calculated between unmethylation and methylation, 18qLOH negative and positive, and DCC alteration negative and positive(total) by Piason's chi-square test

### LOH of 18q locus associated with CIN phenotype in gastric cancer

Among informative cases, the frequencies of 18q LOH at each microsatellite marker were 14/41 (24 %) at D18S35, 17/55 (31 %) at D18S69, and 21/58 (36 %) at D18S58 (the location of each maker is shown in Fig. 1a). Tumors showing LOH at all three, two, and only one of the three microsatellite markers were 4 (4.8 %), 9 (11 %), and 23 (28 %) of 83 informative cases among 98 primary gastric cancers, respectively. Tumors showing LOH in at least one of the three microsatellite markers for 18q LOH were categorized as 18q LOH-positive. By this criterion, 18q LOH-positive cancers were detected in 36 (43 %) of 83 informative cases among 98 primary gastric cancers (Table 2).

Similarly to the *DCC* methylation status, we investigated associations between 18q LOH status and various clinicopathological and genetic features. Among informative cases, the frequency of gastric cancer with distant metastases was higher in 18q LOH-positive gastric cancers compared with 18q LOH-negative cancers (25 vs. 9 %, *P* = 0.041; Table 2). The LOH ratios calculated for the other seven loci were also significantly higher for 18q LOH-positive than for 18q LOH-negative tumors (0.44 vs. 0.10, *P* < .0001; Table 2). According to these results, when a tumor showed a LOH ratio higher than 0, the tumor was categorized as CIN-positive. Furthermore, CIN-positive gastric cancers that were also 18q LOH-positive were significantly more abundant than those that were 18q LOH-negative (74 vs. 38 %, *P* = 0.0017; Table 2). Previous studies have demonstrated that 18q loss is commonly observed in colorectal cancers, and its frequency is correlated with CIN phenotype but inversely correlated with MSI phenotype [29, 30]. As our study and another study demonstrated, this phenomenon was also reproduced for gastric cancers [18].

### Expression and methylation status of *DCC* in gastric cancer cell lines

To assess associations between *DCC* expression status and epigenetic alterations in the *DCC* gene, we examined messenger RNA (mRNA) levels by reverse transcription quantitative polymerase chain reaction (RT-qPCR) using a primer set previously described [31] and examined associations between *DCC* expression and CpG methylation status in the *DCC* promoter region in 10 gastric cancer cell lines (MKN7, N87, MKN74, MKN45, NUGC-2, NUGC-3, NUGC-4, GCIY, OGUM-1, and MKN1) and one normal lung fibroblast cell line (NHLF). All cell lines, except for MKN74, GCIY, OGUM-1, MKN1, and NHLF cells, showed decreased expression of *DCC* gene transcripts, and their promoters were methylated (Fig. 1e). On the other hand, although three cell lines, N87, OGUM-1, and MKN1 showed decreased expression of *DCC* gene transcripts, *DCC* promoters in those cells were not methylated by COBRA. Among the 10 gastric cancer cell lines, only the GCIY cell line expressed *DCC* transcripts at the same level as NHLF cells, but its promoter was methylated. When we categorized a cell line showing aΔC_T_ of more than 15.0 as *DCC* expression-negative and a cell line with aΔC_T_ of 15.0 or less as *DCC* expression-positive, only the GCIY cell line could be categorized as *DCC* expression-positive among the seven gastric cancer cell lines with *DCC* methylation; this finding was statistically non-significant.

### Reduction of DCC expression requires both genetic and epigenetic alterations

**Fig. 2:**
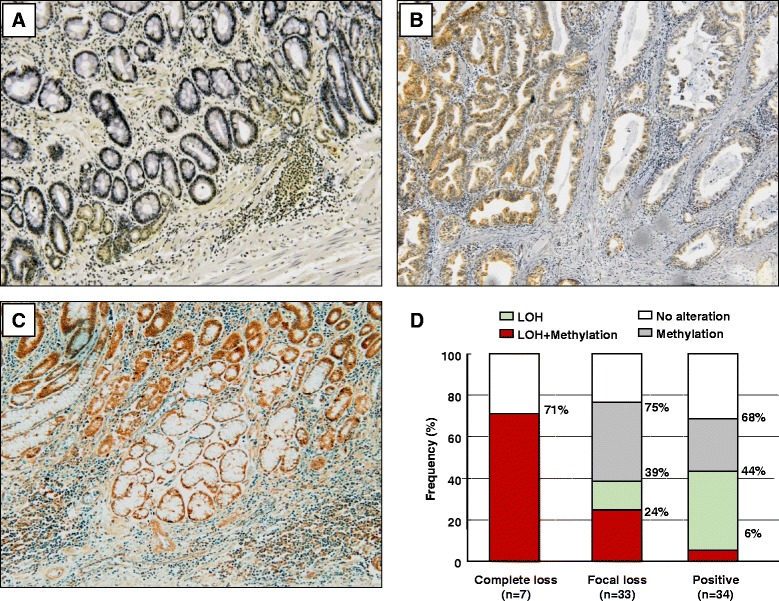
*DCC* promoter methylation and immunohistochemistry analyses. Immunohistochemistry analysis for *DCC* (**a–c**). Nuclei of tumor cells are completely negatively (**a**), focally negatively (**b**), and positively (**c**) stained. (**d**) Association between epigenetic/genetic alteration and *DCC* expression

Next, we investigated the association between DCC protein expression and genetic and epigenetic alterations in the *DCC* gene in 86 gastric cancer specimens. Representative examples of immunohistochemistry (IHC) staining results are shown in Fig. 2a–c. We categorized tumors into the following three groups based upon the IHC results analyzed as a categorical variable: complete loss of DCC expression (8 cases, 9 %; Fig. 2a), focal loss of DCC expression (38 cases, 44 %; Fig. 2b), and positive DCC protein expression (40 cases, 47 %; Fig. 2c). Among 86 gastric cancers, 46 cases (53 %) displayed reduced DCC expression. A previous study reported that reduced DCC expression was observed in a total of 38 % of gastric cancers, and stage T1–T2 tumors maintained a positive DCC expression while it was abolished in T3 tumors [32]. This finding was also reproducible in this study (Additional file 2: Figure S2).

Next, we examined associations between genetic and epigenetic alterations and DCC expression status. Seven of 8 cases with complete loss, 33 of 38 cases with focal loss, and 34 of 40 cases with positive DCC expression were informative for both *DCC* promoter methylation and 18q LOH. We found that among the cancers with complete loss of DCC expression, 5/7 cancers (71 %) demonstrated both *DCC* promoter methylation and 18q LOH. In contrast, 8/33 cancers (24 %) showed focal loss of DCC expression and both methylation and LOH, 5/33 cancers (15 %) showed LOH alone, and 12/33 cancers (36 %) displayed methylation alone. Among the cancers that were positive for DCC expression, only 2/34 cancers (6 %) demonstrated both methylation and LOH, 13/34 (38 %) cancers LOH alone, and 8/34 cancers (24 %) methylation alone (cancers showing both *DCC* methylation and 18q LOH vs. the others, *P* = 0.0048, Pearson’s chi-square test; Fig. 2d). Our data suggest that a reduction in DCC expression may require dense methylation in the promoter CpGs and LOH of the 18q locus, according to the two-hit theory [33].

### Association between clinicopathological features and genetic/epigenetic alterations of *DCC* in gastric cancer

Since both epigenetic and genetic alterations are critical to DCC suppression, we investigated the relationship between epigenetic and genetic alterations in the *DCC* gene with various clinicopathological features. Of 98 gastric cancers, 15 cancers were categorized as non-informative, 25 cancers were categorized as negative for *DCC* alterations, and 58 cancers were categorized as positive for *DCC* alterations. Among clinicopathological features, LOH ratio and CIN phenotype distribution differed significantly between cancers negative and positive for *DCC* alterations (Table 2).

Among the 58 cancers with *DCC* alterations, 17 tumors showed alterations in both *DCC* methylation and 18q LOH, 19 had 18q LOH alone, and 22 cancers exhibited *DCC* methylation alone. The LOH ratio calculated for the other seven loci was significantly highest in gastric cancers with both *DCC* methylation and 18q LOH (mean LOH ratio, 0.44; SD ±0.39) and in cancers with 18q LOH alone (mean LOH ratio, 0.44; SD ±0.34), intermediate in cancers with *DCC* methylation alone (mean LOH ratio, 0.16; SD ±0.18), and lowest in cancers negative for *DCC* alterations (mean LOH ratio, 0.05; SD ±0.10; *P* < 0.0001, Wilcoxon/Kruskal–Wallis test). When we categorized gastric cancers with LOH ratios higher than 0 as CIN-positive, 12/17 gastric cancers (71 %) with both *DCC* methylation and 18q LOH, 13/17 gastric cancers (76 %) with 18q LOH alone, 12/22 gastric cancers (55 %) with *DCC* methylation alone, and 6/25 cancers (24 %) negative for *DCC* alterations were categorized as CIN-positive (*P* = 0.0025, Pearson’s chi-square test). Our data suggest that *DCC* alterations caused by both epigenetic and genetic alterations were significantly associated with gastric cancers exhibiting the CIN phenotype.

### Methylation profiles of *UNC5C* in gastric cancer specimens

**Fig. 3:**
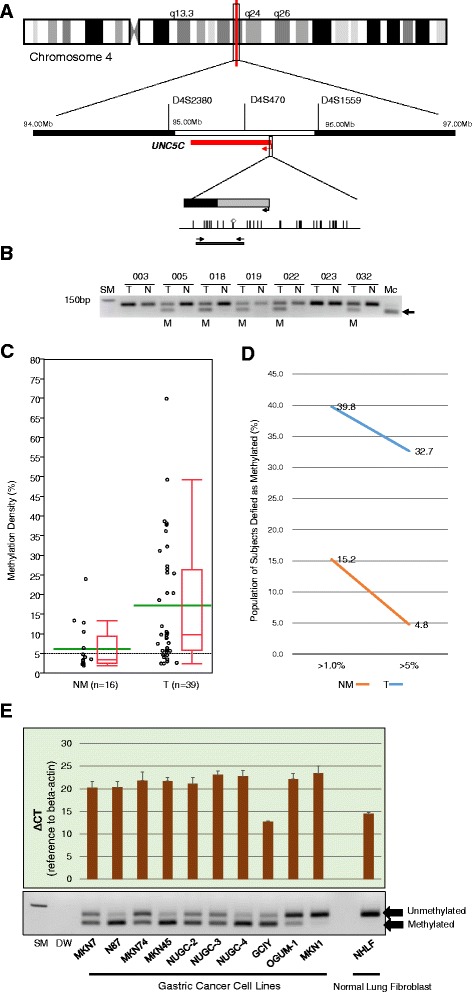
*UNC5C* promoter methylation and 4q LOH analyses. (**a**) Schematic representation of the location of the three LOH probes and *UNC5C* gene promoter regions in chromosome 4. The *red line* denotes the *UNC5C* gene. *Gray and black squares* represent the untranslated and the coding exon 1 regions, respectively; *arrows on the squares* indicate transcriptional starting sites; *vertical lines* indicate CpG sites; *white diamonds* represent the restriction sites for HhaI; *thick horizontal lines* depict the location of COBRA products; *arrows on the thick horizontal lines* denote COBRA primers. (**b**) Representative results of COBRA of *UNC5C. Arrows* indicate methylated alleles; *M* denotes methylation; *U* denotes unmethylation; *Mc* denotes the methylated control; *SM* denotes the size marker. (**c**) Results of *UNC5C* methylation as a continuous variable. In the box plot diagrams, the *horizontal line* within each box represents the median, the *limits* of each box represent the interquartile ranges, and the *whiskers* denote the maximum and minimum values. Each *green bar* represents the mean. *NM* denotes normal mucosa. *T* denotes tumor. (**d**) The frequency of methylation-positivity for cancer and normal tissues according to different thresholds. (**e**) *UNC5C* mRNA expression levels and methylation status in 10 gastric cancer cell lines and a human lung fibroblast cell line. *UNC5C* mRNA expression is observed (lower ΔC_T_) in GCIY and NHLH cell lines. *DW* denotes distilled water

*UNC5C* methylation status was examined in a cohort of 98 gastric cancers and 105 normal gastric mucosa specimens. Location of the *UNC5C* gene and a panel of representative COBRA results are depicted in Fig. 3a–b. We analyzed these results using *UNC5C* methylation levels as continuous variables. We found that 39/98 gastric cancers (40 %) and 16/105 normal gastric mucosa specimens (15 %) displayed more than 1 % methylation in the *UNC5C* promoter. Of the samples that showed more than 1 % methylation in the *UNC5C* promoter, mean methylation levels of *UNC5C* were significantly higher in gastric cancers compared with their corresponding normal gastric mucosa specimens (17.4 % (95 % CI, 12.4–22.4 %] in gastric cancers; 6.3 % (95 % CI, 3.1–9.5 %] in normal gastric mucosa specimens; *P* < 0.0001, Wilcoxon/Kruskal–Wallis test; Fig. 3c–d). Using these results, we optimized a cutoff value of *UNC5C* methylation of 5 % (>5 % methylation as positive and <5 % methylation as negative). Using this cutoff value, 31/98 gastric cancers (32 %) and 5/105 normal gastric mucosa specimens (5 %) were diagnosed as *UNC5C*-methylated. Next, we examined associations between *UNC5C* promoter methylation status and the clinicopathological and genetic features of gastric cancers. *UNC5C* methylation showed a significant association with MSI status. MSI-positive gastric cancers were significantly more frequent in gastric cancers with *UNC5C* methylation compared with those without *UNC5C* methylation (26 vs. 7 %, *P* = 0.013; Table 3). There were no significant associations between *UNC5C* methylation status and any of the other variables.

**Table 3:** Association between epigenetic/genetic alterations of the UNC5C gene and clinicopathological features in gastric cancers

		*UNC5* Methylation Status—% (No.)			4q LOH Status—% (No.)				*UNC5C* Alteration Status—% (No.)						
		Unmethylation	Methylation	*P*	Not informative	Negative	Positive	*P*	Not informative	Negative	Positive	Positive			*P*
											Total	Methylation alone	LOH alone	Both	
		(*n* = 67)	(*n* = 31)		(*n* = 19)	(*n* = 56)	(*n* = 23)		(*n* = 19)	(*n* = 39)	(*n* = 40)	(*n* = 17)	(*N* = 13)	(*N* = 10)	
Age	Mean age (SD)	64.3 (12.6)	66.7 (9.7)	0.49^a^	60.2 (12.6)	65.4 (12.3)	68.3 (8.2)	0.42^a^	60.2 (12.6)	64.8 (13.3)	67.8 (8.9)	67.0 (10.0)	67.2 (8.3)	69.8 (8.2)	0.36^a^
Gender	Female	36 (24)	32 (10)	0.73^b^	47 (9)	36 (20)	22 (5)	0.23^b^	47 (9)	33 (13)	30 (12)	41 (7)	38 (5)	0 (0)	0.75^b^
	Male	64 (43)	68 (21)		53 (10)	64 (36)	78 (18)		53 (10)	67 (26)	70 (28)	59 (10)	62 (8)	100 (10)	
Histology	Diff	48 (32)	52 (16)	0.72^b^	58 (11)	39 (22)	65 (15)	0.036^b^	58 (11)	41 (16)	52 (21)	35 (6)	54 (7)	80 (8)	0.31^b^
	Undiff	520(35)	48 (15)		42 (8)	61 (34)	35 (8)		42 (8)	59 (23)	48 (19)	65 (11)	46 (6)	20 (2)	
Stage	IA/IB	22 (15)	10 (3)	0.17^b^	26 (5)	21 (12)	4 (1)	0.29^b^	26 (5)	28 (11)	5 (2)	6 (1)	8 (1)	0 (0)	0.03^b^
	IIA/IIB	33 (22)	23 (7)		21 (4)	29 (16)	39 (9)		21 (4)	33 (13)	30 (12)	18 (3)	38 (5)	40 (4)	
	IIIA/IIIB/IIIC	31 (21)	52 (16)		37 (7)	38 (21)	39 (9)		37 (7)	28 (11)	48 (19)	59 (10)	31 (4)	50 (5)	
	IV	13 (9)	16 (5)		16 (3)	13 (7)	17 (4)		16 (3)	10 (4)	18 (7)	18 (3)	23 (3)	10 (1)	
T	T1a/1b	18 (12)	6 (2)	0.20^b^	21 (4)	16 (9)	4 (1)	0.49^b^	21 (4)	21 (8)	5 (2)	6 (1)	8 (1)	0 (0)	0.15^b^
	T2	16 (11)	10 (3)		11 (2)	14 (8)	17 (4)		11 (2)	18 (7)	13 (5)	6 (1)	15 (2)	20 (2)	
	T3	22 (15)	39 (12)		26 (5)	25(14)	35 (8)		26 (5)	23 (9)	33 (13)	29 (5)	23 (3)	50 (5)	
	T4a/4b	43 (29)	45 (14)		42 (8)	45 (25)	43 (10)		42 (8)	38 (15)	50 (20)	59 (10)	54 (7)	30 (3)	
N	N0	31 (21)	16 (5)	0.06^b^	32 (6)	32 (18)	9 (2)	0.19^b^	32 (6)	38 (15)	13 (5)	18 (3)	15 (2)	0 (0)	0.03^b^
	N1	39 (26)	26 (8)		42 (8)	30 (17)	39 (9)		42 (8)	33 (13)	33 (13)	24 (4)	38 (5)	40 (4)	
	N2	19 (13)	35 (11)		11 (2)	25 (14)	38 (8)		11 (2)	21 (8)	35 (14)	35 (6)	31 (4)	40 (4)	
	N3	10 (7)	23 (7)		16 (3)	13 (7)	17 (4)		16 (3)	8 (3)	20 (8)	24 (4)	15 (2)	20 (2)	
Distant metastasis	Negative	87 (58)	84 (26)	0.72^b^	84 (16)	88 (49)	83 (19)	0.57^b^	84 (16)	90 (35)	39	94 (16 )	100 (13)	100 (10)	0.35^b^
	Positive	13 (9)	16 (5)		16 (3)	13 (7)	17 (4)		16 (3)	10 (4)	0	6 (1)	0 (0)	0 (0)	
MSI	MSI	7 (5)	26 (8)	0.013^b^	21 (4)	14 (8)	4 (1)	0.21^b^	21 (4)	10 (4)	5	24 (4)	0 (0)	10 (1)	0.75^b^
	Non-MSI	93 (62)	74 (23)		79 (15)	86 (48)	96 (22)		79 (15)	90 (35)	35	76 (13)	100 (13)	90 (9)	
LOH Ratio	Mean ratio (SD)	0.21 (0.28)	0.31 (0.35)	0.19^a^	0.32 (0.36)	0.15 (0.25)	0.40 (0.29)	<0.0001^a^	0.32 (0.36)	0.11 (0.22)	0.33 (0.30)	0.24 (0.29)	0.37 (0.25)	0.45 (0.36)	0.0001^a^
CIN*	Positive	49 (32)	58 (18)	0.42^b^	58 (11)	37 (20)	83 (19)	0.0003^b^	58 (11)	30 (11)	28	53 (9 )	85 (11)	80 (8)	0.004^b^
	Negative	51 (33)	42 (13)		42 (8)	63 (34)	17 (4)		42 (8)	70 (26)	12	47 (8)	15 (2)	20 (2)	
*KRAS*/*BRAF*/*PIK3CA*	Mutant	6 (4)	13 (4)	0.24^b^	5 (1)	13 (7)	0 (0)	0.08^b^	5 (1)	8 (3)	4	24 (4)	0 (0)	0 (0)	0.72^b^
	Wild	94 (63)	87 (27)		95 (18)	88 (49)	100 (23)		95 (18)	92 (36)	36	76 (13)	100 (13)	100 (10)	
H.pyroli	Positive	72 (48)	71 (22)	0.95^b^	68 (13)	70 (39)	78 (18)	0.44^b^	68 (13)	67 (26)	9	24 (4)	15 (2)	30 (3)	0.28^b^
	Negative	28 (19)	29 (9)		32 (6)	30 (17)	22 (5)		32 (6)	33 (13)	31	76 (13)	85 (11)	70 (7)	

*Two cases are not informative of CIN status

^a^ *P* value were calculated between unmethylation and methylation, 4qLOH negative and positive, and UNC5C alteration negative and positive(total) by Wilcoxon/Kruskal–Wallis test

^b^ *P* values were calculated by Pearson’s chi-square test

### Expression and methylation status of *UNC5C* in gastric cancer cell lines

Before examining UNC5C expression and methylation status in the *UNC5C* promoter region in the gastric cancer cell lines, we assessed the expression status of five splicing variants of *UCNC5C* mRNA (UNC5C-001, 002, 003, 004, and 201) by RT-PCR (Additional file 3: Figure S3). When analyzing five splicing variants of *UCNC5C* messenger RNA in 10 gastric cancer cell lines (MKN7, N87, MKN74, MKN45, NUGC-2, NUGC-3, NUGC-4, GCIY, OGUM-1, and MKN1) and one normal lung fibroblast cell line (NHLF), we found that only two cell lines, GCIY and NHLF, expressed all five splicing variants of *UCNC5C* messenger RNA. In nine gastric cancer cell lines, there was no expression of any of the five splicing variants of *UCNC5C* mRNA (Additional file 4: Figure S4). Thus, further examination of mRNA expression levels by RT-qPCR was performed using the *UNC5C* 001–004 primer set. Among the 10 gastric cancer cell lines, only the GCIY cell line exhibited increased *UNC5C* mRNA expression levels (lowerΔC_T_, Fig. 3e). When we categorized UNC5C mRNA expression status by RT-PCR results (Additional files 4 and 5: Figures S4 and S5), 93 % (13/14) of *UNC5C* methylated cell lines lacked *UNC5C* mRNA expression, whereas 33 % (1/3) of *UNC5C* unmethylated cell lines lacked *UNC5C* mRNA expression (*P* = 0.01, Pearson’s chi-square test). Thus, *UNC5C* gene transcript expression was significantly associated with promoter methylation level. Additionally, we tried to perform IHC staining for UNC5C protein expression. Unfortunately, we were unable to analyze UNC5C protein expression due to lack of appropriate antibodies.

### LOH of the 4q locus associated with CIN phenotype in gastric cancer

The frequencies of 4q21–23 LOH at each microsatellite marker for *UNC5C* were 29 % (11/38 informative cases) at D4S2380, 23 % (10/44) at D4S470, and 28 % (12/43) at D4S1559 (location of each maker is shown in Fig. 3a). Tumors showing LOH at all three, two, and only one of the three microsatellite markers amounted to 3 (3.8 %), 5 (6.3 %), and 15 (20 %) of 79 informative cases among 98 primary gastric cancers, respectively. We defined 4q LOH-positive tumors as those showing LOH on at least one of the three microsatellite markers. Tumors showing 4q LOH were found in 23 (29 %) of 79 informative cases among 98 primary gastric cancers (Table 3). We found that differentiated adenocarcinomas were significantly more frequently observed in cancers with 4q LOH (65 % in 4q LOH-positive cancers vs. 39 % in 4q LOH-negative ones, *P* = 0.036).

Similar to 18q LOH, we found a strong correlation between 4q LOH and CIN phenotype. The LOH ratio calculated for the remaining seven loci was significantly higher in gastric cancers with 4q LOH compared with those without 4q LOH (0.40 vs. 0.15, *P* < 0.0001; Table 3). According to these results, when a tumor showed a LOH ratio higher than 0, the tumor was categorized as CIN-positive, and CIN-positive gastric cancers were significantly more frequent in the presence of 4q LOH (83 % in 4q LOH-positive tumors vs. 37 % in 4q LOH-negative ones, *P* = 0.0003; Table 3). While there were no significant associations among any of the other variables, all *KRAS*/*PIK3CA* mutations were found in 4q LOH-negative gastric cancers with a non-significant difference (*P* = 0.08).

### Association between clinicopathological features and genetic/epigenetic alterations of *UNC5C* in gastric cancer

Next, we investigated the relationship between *UNC5C* alterations and clinicopathological features. Of 98 gastric cancers, 19 cancers were categorized as non-informative, 39 cancers were categorized as negative for *UNC5C* alterations, and 40 cancers as positive for *UNC5C* alterations. Cancers with *UNC5C* alterations were more frequently observed in advanced stages (37 % (14/38) for stages I and II vs. 63 % (26/41) for stages III and IV, *P* = 0.02, Pearson’s chi-square test) and in advanced categories for lymph node metastasis (25 % (5/20) for N0 vs. 59 % (35/59) for N1–N3, *P* = 0.008, Pearson’s chi-square test).

Among the 40 cancers with *UNC5C* alterations, 10 cancers showed alterations both in terms of *UNC5C* methylation and 4q LOH, 13 cancers showed 4q LOH alone, and 17 cancers showed *UNC5C* methylation alone. The LOH ratio calculated for the remaining seven loci was significantly higher in cancers with both *UNC5C* methylation and 4q LOH (mean LOH ratio, 0.45; SD ±0.36) and in those with 4q LOH alone (0.37 ± 0.25), intermediate in cancers with *UNC5C* methylation alone (0.24 ± 0.29), and lowest in cancers negative for *UNC5C* alterations (0.11 ± 0.22, *P* = 0.0002, Wilcoxon/Kruskal–Wallis test). When we categorized the cancers that showed LOH ratios higher than 0 as CIN-positive, 8/10 gastric cancers (80 %) with both *UNC5C* methylation and 4q LOH, 11/13 gastric cancers (85 %) with 4q LOH alone, 9/17 gastric cancers (53 %) with *UNC5C* methylation alone, and 11/39 cancers (30 %) negative for *UNC5C* alterations were categorized as CIN-positive (*P* = 0.001, Pearson’s chi-square test). Similar to the DCC alterations in gastric cancer, our data highlight that *UNC5C* alterations caused by both epigenetic and genetic events were significantly associated with CIN-positive gastric cancers.

### Cumulative loss of netrin-1 receptors accrues with gastric cancer progression

**Fig. 4:**
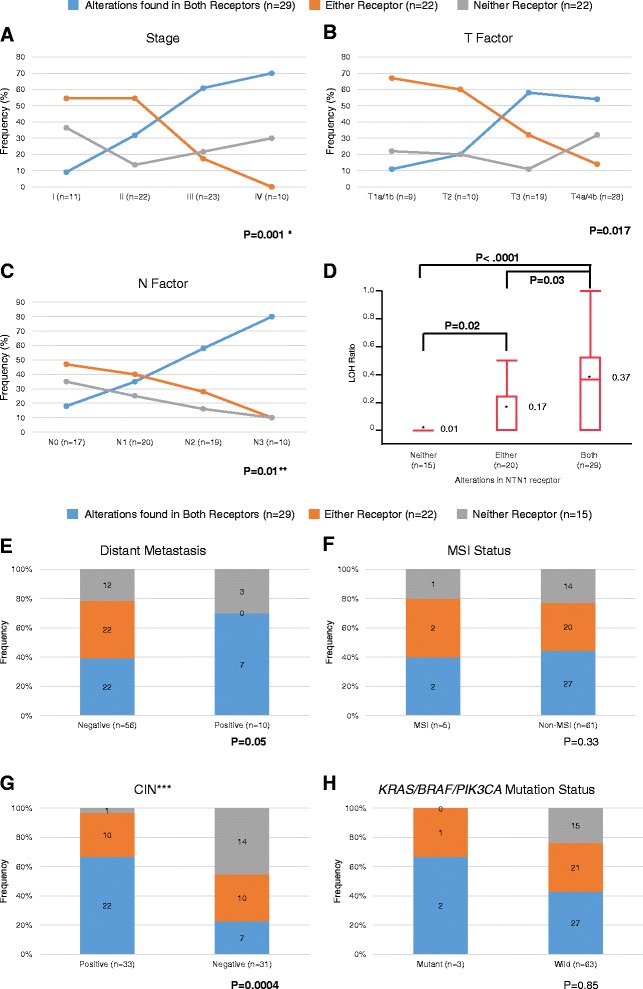
Association between alteration patterns in netrin-1 receptors and clinicopathological features in gastric cancers. Correlation between alterations in netrin-1 receptors and TNM stage (**a**), depth of invasion (**b**), and degree of regional lymph node metastasis (**c**); association between alteration patterns in netrin-1 receptors and LOH ratio (**d**), presence of distant metastasis (**e**), MSI status (**f**), CIN phenotype (**g**), and *KRAS*/*BRAF*/*PIC3CA* mutation status (**h**). *The *P* value in panel A was calculated between Stage I/II *vs*. III/IV by Pearson’s chi-square test. ** The *P* value in panel **c** was calculated between N0 vs. N1–N3 by Pearson’s chi-square test. In Panel (**d**), the *horizontal line* within each box represents the median, the *limits* of each box represent the interquartile ranges, and the *whiskers* are the maximum and minimum values in the box plot diagrams. *Asterisks* and the *numbers* denote the mean value of the LOH ratio. Pairwise comparisons for each of the subgroups in panel (**d**) were performed by a non-parametric multiple comparison method using the Steel–Dwass test. ***Two cases could not be evaluated for CIN phenotype

Because UNC5C and DCC both serve as dependence receptors for netrin-1, we investigated whether defects in these receptors accumulate in a systematic or stochastic manner during the progression of gastric carcinoma. Therefore, we looked for associations between *UNC5C* and/or *DCC* defects and TNM stage in the 98 gastric cancers that were informative for both *UNC5C* and *DCC* genetic/epigenetic results (Fig. 4 and Additional file 6: Table S1). Concurrent alterations in the *DCC* and *UNC5C* genes were observed significantly more commonly in advanced stages (64 % (21/ 33) for stages III and IV) than in earlier-stage cancers (24 % (8/33) for stages I and II, *P* = 0.001, Pearson’s chi-square test; Fig. 4a). By stratifying gastric cancers based on individual defects in either *UNC5C* or *DCC* and their relationship with tumor stage, *UNC5C* alterations were found in 18 % (2/11 informative cases), 48 % (12/25 informative cases), 63 % (19/30 informative cases), and 64 % (7/11 informative cases) of stages I, II, III, and IV cancers, respectively, showing that *UNC5C* alterations gradually developed according to the progression of the TNM stage (Table 3). On the other hand, *DCC* alterations were constantly observed with a high frequency in all TNM stages; hence, *DCC* alterations were found in 63 % (10/16 informative cases), 76 % (19/25 informative cases), 66 % (19/29 informative cases), and 77 % (10/13 informative cases) of stages I, II, III, and IV cancers, respectively (Table 2). Therefore, this differential feature found for each gene taken into consideration indicates that cumulative alterations of netrin-1 receptors are associated with gastric cancer progression. With respect to the factors that determine TNM classification, a cumulative loss of netrin-1 receptors was more strongly associated with the degree of regional lymph node metastasis (N factor, Fig. 4c) compared with the tumor status of T factors (Fig. 4b). Interestingly, gastric cancers with defects in either *UNC5C* or *DCC* did not show distant metastasis (Fig. 4e), suggesting that the cumulative alterations of netrin-1 receptors was a late event in gastric cancer progression, significantly associated with CIN-positive gastric cancers through increasing the LOH ratio (Fig. 4d, g) rather than MSI and mutational status (Fig. 4f). On the other hand, there were no significant associations between cumulative loss of netrin-1 receptors and any other clinicopathological variables (Additional file 7: Figure S6).

## Discussion

This study investigated the molecular events responsible for the abrogation of the netrin pathway in gastric cancer and the role played by the two dependence receptors, *DCC* and UNC5C. We analyzed 98 gastric cancers and 105 adjacent normal mucosa specimens. We found that the frequency of gastric cancers with concurrent alterations in the *DCC* and *UNC5C* genes increased in a stage-dependent manner. Upon stratifying gastric cancers based on defects in either *DCC* or *UNC5C* and on their relationship with tumor stage, we found that *DCC* alterations were consistently observed in all TNM stages with a high frequency: 10 of 16 (63 %) of stage I, 19 of 25 (76 %) of stage II, 19 of 29 (66 %) of stage III, and 10 of 13 (77 %) of stage IV cancers (Table 2). Meanwhile, *UNC5C* alterations gradually increased according to the progression of the TNM stage and were found in 2/11 (18 %) of stage I, 12 of 25 (48 %) of stage II, 19 of 30 (63 %) of stage III, and 7 of 11 (64 %) of stage IV cancers (Table 3). Both *DCC* and *UNC5C* were inactivated in 97 % of CIN-positive gastric cancers and in 55 % of CIN-negative gastric cancers, and these alterations occurred through genetic and epigenetic processes. These data provide novel evidence that the timing of molecular alterations in *DCC* and *UNC5C* is not random, because *DCC* inactivation occurs through all tumor stages, whereas *UNC5C* inactivation accrues gradually during multistep gastric carcinogenesis.

Cells expressing netrin-1 receptors can send a survival signal when they are engaged by netrin-1. On the other hand, these receptors will send a death signal when they are disengaged [34]. Thus, a loss of netrin-1 receptors on tumor cells represents a loss of dependence receptors that are capable of mediating apoptosis, resulting in enhanced tumor cell survival [35].

The netrin-1 receptor, *DCC*, was discovered as a putative tumor suppressor gene in colorectal cancer [21]. *DCC* is located on chromosome 18q, which is the most common deleted chromosomal region in colorectal cancer as well as gastric cancer [13, 36–38, 12, 14]. The tumor-suppressor role for *DCC* has been questioned in studies that failed to show a clear malignant phenotype in *DCC* knockout mouse models [20]. However, recent studies have also challenged this hypothesis and have suggested a role for *DCC* in suppressing tumor growth and metastasis [24, 25]. Recent indications that *DCC* serves as a dependence receptor for netrin-1 have renewed the hypothesis that *DCC* functions as a pro-apoptotic growth suppressor when not bound to its ligand [34, 39, 12, 10]. In the gastrointestinal tract, netrin-1 has an important role in the maintenance and renewal of the intestinal epithelium by regulating cell survival or cell death through its interaction with its receptors, *DCC* and UNC5C [24, 34, 39]. In line with previous studies [11, 8], in this study, we demonstrated that methylation-induced silencing of *DCC* as well as allelic loss of 18q was critical to loss of *DCC* expression. Thus, reduction of *DCC* expression may require dense CpG promoter methylation and LOH of the 18q locus according to the two-hit theory as a common behavior of tumor suppressor genes [33].

The other netrin-1 receptors, UNC5A, UNC5B, and UNC5C, were also discovered as putative tumor suppressor genes in various tumors [26, 27]. Among them, a twofold downregulation of UNC5C expression compared with corresponding normal tissue was observed in approximately 70 % of gastric cancer cases [26]. Therefore, we focused on UNC5C that may play a more critical role for gastric carcinogenesis. Additionally, it was suggested that the loss of UNC5C was caused by allelic losses of chromosome 4q, and mutations were rarely observed [26]. Allelic losses at the 4q locus have been reported previously in several human cancers, with frequencies ranging from 23–39 % [26, 32, 18]. In accordance with previous studies, in the present study, the frequencies of allelic loss at 4q21–23 LOH were 29 % (11/38 informative cases) on D4S2380, 23 % (10/44) on D4S470, and 28 % (12/43) on D4S1559. Finally, gastric cancers demonstrating allelic loss at 4q were found in 29 % (23/79) of the informative cases. Another mechanism underlying the loss of UNC5C in human cancers is represented by epigenetic alterations. Indeed, we previously reported that UNC5C was silenced by dense methylation of its promoter CpG islands in colorectal cancer [28]. As is the case in colorectal cancer, our results demonstrated that 11/12 gastric cancer cell lines (Fig. 3e and Additional files 3 and 4: Figures S3–S4) lacking *UNC5C* expression showed dense methylation in the *UNC5C* promoter. In clinical specimens, 31/98 gastric cancers (32 %) and 5/105 normal gastric mucosa specimens (5 %) exhibited *UNC5C* methylation. Therefore, we performed UNC5C IHC on the clinical specimens to examine the two-hit theory in which aberrant promoter methylation and allelic losses were the key factors determining lack of *DCC*. However, we were unable to perform UNC5C IHC on the clinical specimens because of the lack of an appropriate antibody for tissue staining.

Because both *DCC* and *UNC5C* share the same netrin ligand and are colocalized in the gut [24, 23, 26, 40], we hypothesized that solitary inactivation of either *DCC* or *UNC5C* may not be sufficient to promote tumor development in the stomach. In this study, we found that 97 % of gastric cancers with CIN and 55 % of those without CIN showed simultaneous alterations in both *DCC* and *UNC5C*, supporting our hypothesis that inactivation of both receptors may be required in the development of gastric cancer. Our finding that dysregulation of *DCC* predominantly occurs in the early phase of gastric cancer whereas *UNC5C* alterations occur later suggests that inactivation of these receptors is not a random process but occurs in a statistically predictable, sequential manner.

*H. pylori* in the human gastric mucosa is a well-known inducer of chronic inflammation and gastric cancers and is associated with a high incidence of aberrant DNA methylation [7, 41, 30, 29]. So, we detected *H. pylori* infection by recovering the *cagA* repeat sequence from gastric cancer specimens as well as normal gastric mucosa samples. Of 98 cancers, 70 cancers were positive for the *cagA* sequence. However, there was no association between the presence of the *cagA* sequence in cancer tissue and clinicopathological and molecular features, specifically not *DCC* nor *UNC5C* methylation incidence. Conversely, with respect to normal counterpart gastric mucosa, a total of 102 normal gastric mucosa samples were available for analyzing the presence of the *cagA* sequence in this study. Of 102 gastric mucosa samples, the *cagA* sequence was successfully recovered in 79 (74 %). Interestingly, when we defined *DCC* and *UNC5C* methylation at 1 % or more as a continuous variable (i.e., >1.0 % methylation as methylation-positive (methylated) and <1.0 % methylation as methylation-negative (unmethylated)), only *UNC5C* methylation was significantly associated with the presence of the *cagA* sequence in normal gastric mucosa (data not shown), suggesting that inflammatory processes associated with *H. pylori* infection causes aberrant methylation in *UNC5C* promoter CpGs but not in the *DCC* promoter. Thus, our results suggested that *H. pylori* infection does not induce aberrant methylation randomly but in target loci by a particular signal cascade. To address questions underlying *H. pylori* infection and epigenetic changes, further investigations are warranted.

## Conclusions

We provide previously unrecognized and novel evidence that most gastric cancers, particularly those with CIN, possess alterations in both *DCC* and *UNC5C* receptors. Such alterations are apparent in the early stages and continue to escalate in both receptor types with disease progression, emphasizing the importance of this growth regulatory pathway in gastric carcinogenesis.

## Methods

### Primary gastric specimens

We collected tissue specimens of primary gastric cancer and matched normal gastric mucosa from 105 patients who had undergone surgery at the Okayama University Hospital (Okayama, Japan). Of 105 gastric cancer patients, seven did not have sufficient tumor tissue for analysis. Thus, in this study, a total of 98 gastric cancer specimens and 105 matched normal gastric mucosa tissues were analyzed. All normal gastric mucosa specimens were obtained from sites adjacent to the tumor but at least 5 cm away from the tumor site. All patients provided written informed consent, and the study was approved by the ethics committee of the Okayama University Hospital. All patients also gave informed consent for usage of their data for future analyses. The histological diagnosis was established according to the World Health Organization International Histological Classification of tumors, with subclassification into two histological categories: differentiated type (well and moderately differentiated tubular adenocarcinoma) and undifferentiated type (poorly differentiated adenocarcinoma and mucinous adenocarcinoma). The pathological stage was determined according to the International Union Against Cancer TNM classification (Seventh edition).

### Cell lines

A total of 12 human gastric cancer cell lines (MKN7, N87, MKN74, MKN45, NUGC-2, NUGC-3, NUGC-4, GCIY, OGUM-1, MKN1, MKN28, and KATO III), three human colorectal cancer cell lines (SW48, SW480, and SW837), one human colon fibroblast cell line (CCD18Co), and one human lung fibroblast cell line (NHLF) were purchased from American Type Culture Collections (Manassas, VA, USA), Japanese Collection of Research Bioresources Cell Bank (Ibaragi, Osaka, Japan), Health Science Research Resources Bank (Chiyoda-ku, Tokyo, Japan), RIKEN (Wako, Saitama, Japan), or Cambrex Bio Science Walkersville, Inc. (East Rutherford, NJ, USA). All cell lines were cultured in appropriate culture medium supplemented with 10 % fetal bovine serum, penicillin (100 IU/mL), and streptomycin (100 μg/mL) at 37 °C in a humidified incubator with 5 % CO_2_.

### DNA and RNA extraction

Genomic DNA from the cell lines was extracted using QIAamp DNA Mini Kit (Qiagen, Valencia, CA, USA). All gastric cancers and normal gastric mucosa samples were fresh-frozen tissue specimens, from which DNA was extracted using standard procedures that included proteinase-K digestion and phenol–chloroform extraction. Total RNA from six cultured cell lines (MKN28, KATO III, SW48, SW480, SW837, and CCD18Co) was obtained using the TRIzol reagent (Invitrogen Life Technologies Inc., Carlsbad, CA, USA). Total RNA from 10 gastric cancer cell lines (MKN7, N87, MKN74, MKN45, NUGC-2, NUGC-3, NUGC-4, GCIY, OGUM-1, and MKN1) and a normal lung fibroblast cell line, NHLF, was isolated using the RNeasy Mini Kit (Qiagen) according to the manufacturer’s instructions.

### Reverse transcription polymerase chain reaction

The first-strand complementary DNA synthesis was performed using the Moloney murine leukemia virus reverse transcriptase (Invitrogen Life Technologies Inc.) and miScript II RT Kit (Qiagen) with a total of 1.0 μg RNA. RT-PCR was performed using specific primer pairs for *UNC5C* (including primers for the detection of splicing variants), *DCC*, and *beta*-*actin* (Additional file 8: Table S2). The PCR products were electrophoresed on a 3.5 % agarose gel. By using the *UNC5C* 001–004-, *DCC*-, and *beta*-*actin*-specific primer pairs, expression of *UNC5C* and *DCC* mRNA was also determined by RT-qPCR using the SsoAdvanced Universal SYBR Green Supermix on the LightCycler 480 (Roche Diagnostics). The expression level of each target gene was analyzed based on the ΔC_T_ method, and *beta*-*actin* was used as an endogenous control to normalize the amount of total RNA in each sample.

### Bisulfite modification and combined bisulfite restriction analysis

Bisulfite modification of genomic DNA from cell lines and clinical specimens was performed as described previously. The methylation status of the *DCC* and *UNC5C* promoters in gastric tissues and cell lines was analyzed by combined bisulfite restriction analysis (COBRA, Additional file 8: Table S2). COBRA was carried out in a 24.0-μL PCR reaction containing 12.0 μL of HotStarTaq Master Mix (Qiagen) and 0.4 μM of each primer. PCR products were digested with a restriction enzyme HhaI (New England Biolabs Inc., Ipswich, MA, USA) at 37 °C overnight. PCR products were electrophoresed on a 3.0 % agarose gel. The percentage of methylated HhaI sites were calculated by determining the ratio between the HhaI-cleaved PCR product and the total amount of PCR product loaded.

### LOH analyses and definition of CIN phenotype

A set of three polymorphic microsatellite markers per gene was used to determine LOH at chromosomes 18q21 for *DCC* and 4q21–23 for *UNC5C* (Additional file 8: Table S2). PCR amplifications were performed on genomic DNA templates from both tumor and normal mucosa tissue using fluorescently labeled primers. PCR products were electrophoresed on an ABI 310R Genetic Analyzer and analyzed by GeneMapper fragment analysis software (Applied Biosystems, Foster City, CA, USA). When comparing the signal intensities of the individual markers in the tumor DNA with that of the corresponding normal DNA, a reduction by at least 40 % of the signal intensity was considered indicative of LOH.

In addition, to examine the association between netrin-1 receptor disorders and CIN phenotype, we analyzed additional seven sets of polymorphic microsatellite sequences that are tightly linked to known tumor suppressor genes and DNA mismatch repair genes, including the *MYCL* locus on 1p34 (*MYCL*), the *hMSH2* locus on 2p16 (D2S123), the *APC* locus on 5q21 (D5S346, D5S107), the *UNC5D* locus on 8p12 (D8S87), and the *p53* locus on 17p13 (D17S250, TP53) [31]. Of 98 gastric cancer patients, 96 patients displayed at least one marker informative of LOH status. Hence, since two patients turned out non-informative for LOH at all seven microsatellite sequences, further analyses were performed only on the 96 informative cases. CIN phenotype classification was performed by calculating the LOH ratio of the informative markers of the seven polymorphic microsatellite sequences, independently from the *UNC5C* and *DCC* loci. When a tumor showed a LOH ratio higher than 0, the tumor was categorized as CIN-positive.

### MSI analysis and definition of MSI phenotype

MSI status was analyzed for all 98 gastric cancer patients using three mononucleotide repeat markers (BAT26, NR21, and NR27) as described previously [33]. When at least one or more mononucleotide repeat markers displayed microsatellite instability, tumors were defined to have an MSI phenotype, and tumors without MSI in the three mononucleotide repeat markers were defined to have a non-MSI phenotype.

### *KRAS*, *BRAF*, and *PIK3CA* mutation analyses

*KRAS* and *BRAF* mutation status was analyzed in all 98 patients as described previously [35]. In addition, *PIK3CA* exon 9 and 20 mutation status was also analyzed by direct sequencing. PCR and sequencing were performed using *PIK3CA* exon 9- and 20-specific primer pairs (Additional file 8: Table S2). PCR products were electrophoresed on an ABI 310R Genetic Analyzer.

### Detection of *H. pylori*

To determine *H. pylori* infection status, we recovered the Glu-Pro-Ile-Tyr-Ara (EPIYA) repeat sequence in the *cagA* protein, which binds to the Src homology 2 domain-containing protein tyrosine phosphatase, SHP-2, on gastric epithelial cells. The *cagA* was recovered by PCR amplification performed on genomic DNA templates from tumor tissues. We modified the primer design to develop PCR products shorter than the PCR products described previously [42, 43]. PCR was carried out in a 24-μL PCR reaction containing 12 μL of HotStarTaq Master Mix and 0.4 μM of each primer (Additional file 8: Table S2). The PCR products were electrophoresed on a 3 % agarose gel.

### Immunohistochemical analysis

A total of 89 gastric cancers from 98 patients were available for IHC staining for *DCC* protein expression analysis. Staining was carried out manually with formalin-fixed paraffin-embedded tissues. Thin (5 μm) sections of representative blocks were deparaffinized and dehydrated using gradient solvents. Following antigen retrieval in citrate buffer (pH 6.0), endogenous peroxidase was blocked with 3 % H_2_O_2_. Thereafter, slides were incubated overnight in the presence of a purified mouse anti-human *DCC* monoclonal antibody (clone G97–449, Pharmingen, San Diego, CA, USA; dilution 1:100). A further incubation was carried out with a secondary antibody and the avidin-biotin-peroxidase complex (Vector Laboratories, Burlingame, CA, USA) and then incubated with biotinyltyramide followed by streptavidin-peroxidase. Diaminobenzidine was used as a chromogen, and hematoxylin as a nuclear counterstain. Tissue sections with obvious nuclear staining were considered positive. The only foci of neoplasia that were scored as negative were those for which there was definite evidence of unspecific positively staining admixed or surrounding non-neoplastic cells such as normal colonic mucosal cells, lymphocytes, or stromal cells.

### Statistical analysis

Statistical analyses were performed using JMP software (version 10.0; SAS Institute Inc., Cary, NC, USA). First, *DCC* and *UNC5C* methylation levels were analyzed as continuous variables, as were computed means, standard errors of the means, and standard distributions. Next, the methylation status of the *DCC* and *UNC5C* promoter was analyzed as a categorical variable (positive, methylation level >5 %; negative, methylation level < 5 %). Differences in frequencies were evaluated by Pearson’s chi-square test or the Wilcoxon/Kruskal–Wallis test. Whenever the Kruskal–Wallis test indicated differences among these subgroups, further pairwise comparisons for each of the subgroup was performed by a non-parametric multiple comparison method using the Steel–Dwass test. All reported probability (*P*) values were two-sided, and a *P* value of less than 0.05 was considered statistically significant.

## Additional files

Additional file 1: Figure S1. Examples of *KRAS* mutation and *H*. *pylori* cagA analysis. (A) An example of *KRAS* codon 12 and 13 direct sequencing analysis. This case showed both codon 12 and 13 mutations. (B) To detect *H. pylori* infection, EPIYA repeat sequences in the cagA protein were recovered from clinical materials. We found two types of EPIYA repeat sequences, one was 159 bp and the other was 261 bp. SM denotes the size marker; P and N denote positivity and negativity of EPIYA repeat sequences, respectively. Additional file 2: Figure S2. Association between DCC IHC status and T factors. Additional file 3: Figure S3. Splicing variants of *UNC5C* mRNA and location of the primers which can distinguish expression status of the splicing variants. Additional file 4: Figure S4. Expression status of splicing variants of *UNC5C* mRNA and association of *UNC5C* methylation for 10 gastric cancer cell lines. Ten gastric cancer cell lines and NHLF cells were analyzed for mRNA expression by RT-PCR of *UNC5C* splicing variants and *beta*-*actin* genes. The lowest panel illustrates the methylation profile obtained from COBRA. Additional file 5: Figure S5. Association of *UNC5C* methylation and loss of *UNC5C* mRNA expression in gastric cancer and colorectal cancer cell lines. Two gastric cancer cell lines, three colorectal cancer cell lines, and CCD18Co cells were analyzed for mRNA expression by RT-PCR of *UNC5C* and *beta*-*actin* genes. The lowest panel illustrates the methylation profile obtained from COBRA. Additional file 6: Table S1. Association between alterations in netrin-1 receptors and clinicopathological features in gastric cancers. Additional file 7: Figure S6. Association between alteration patterns in netrin-1 receptors and clinicopathological features in gastric cancers. Correlation between alterations in netrin-1 receptors and gender (A), histology (B), *H. pylori* cagA expression status (C), and (D) age. In the box plot diagrams, the horizontal line within each box represents the median, the limits of each box are the interquartile ranges, and the whiskers denote the maximum and minimum values. Asterisks plus numbers denote mean age at surgery. Additional file 8: Table S2. Primer sequences.
