# Supplementary material for: Genetic and epigenetic alterations of netrin-1 receptors in gastric cancer with chromosomal instability
Source: Clin Epigenetics. 2015 Jul 23;7(1):73. doi: 10.1186/s13148-015-0096-y (PMC4511994; doi:10.1186/s13148-015-0096-y)
Supplement: Additional file 1: Figure S1. — Examples of KRAS mutation and H. pylori cagA analysis. (A) An example of KRAS codon 12 and 13 direct sequencing analysis. This case showed both codon 12 and 13 mutations. (B) To detect H. pylori infection, EPIYA repeat sequences in the cagA protein were recovered from clinical materials. We found two types of EPIYA repeat sequences, one was 159 bp and the other was 261 bp. SM denotes the size marker; P and N denote positivity and negativity of EPIYA repeat sequences, respectively. [file 13148_2015_96_MOESM1_ESM.pptx]

## Slide 1
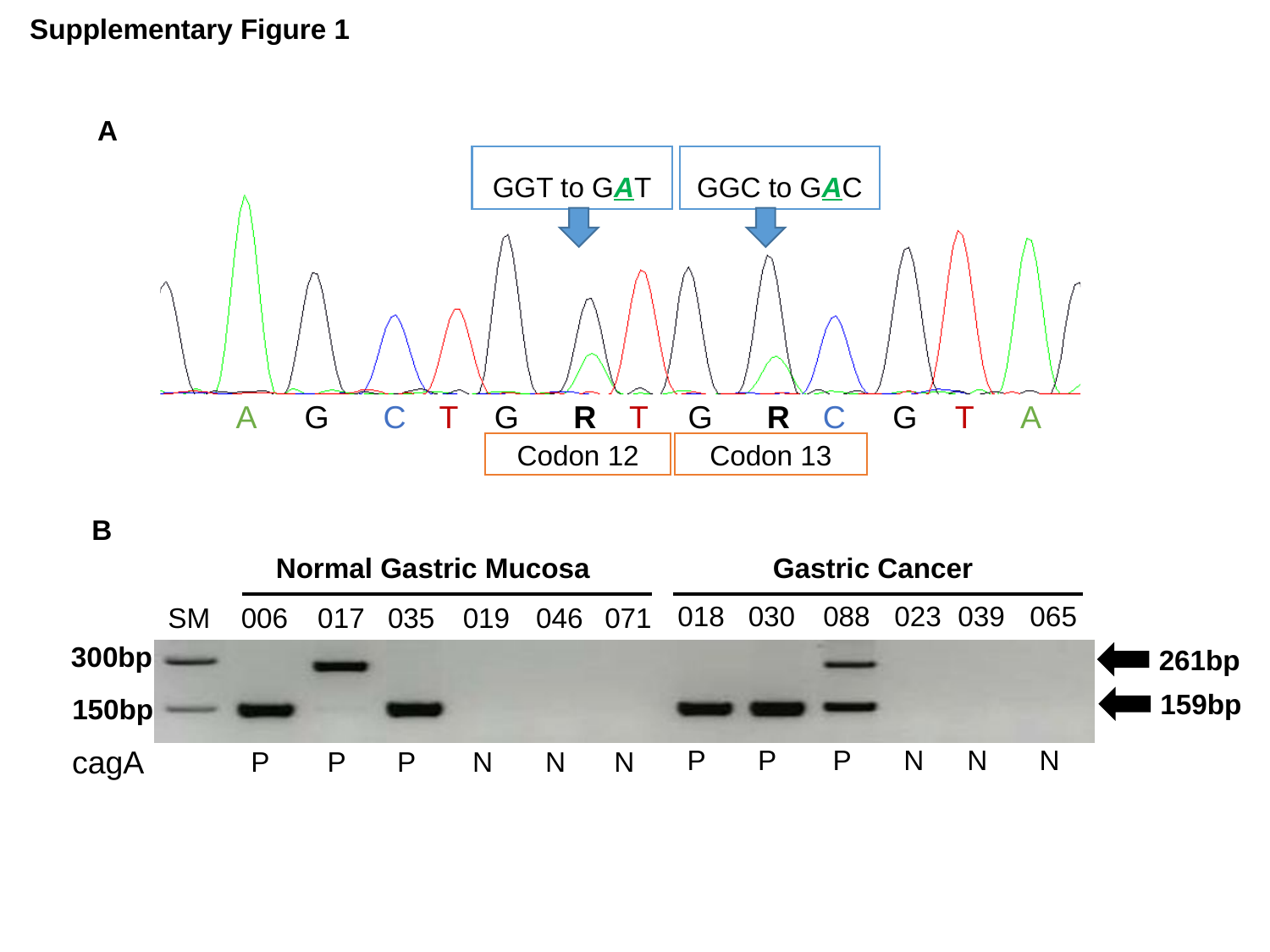

Supplementary Figure 1
A
GGT to GAT
GGC to GAC
A
G
C
T
G
R
T
G
R
C
G
T
A
Codon 12
Codon 13
B
Normal Gastric Mucosa
Gastric Cancer
018
030
088
023
039
065
SM
006
017
035
019
046
071
300bp
261bp
159bp
150bp
cagA
P
P
P
N
N
N
P
P
P
N
N
N
