# Supplementary material for: Genetic and epigenetic alterations of netrin-1 receptors in gastric cancer with chromosomal instability
Source: Clin Epigenetics. 2015 Jul 23;7(1):73. doi: 10.1186/s13148-015-0096-y (PMC4511994; doi:10.1186/s13148-015-0096-y)
Supplement: Additional file 2: Figure S2. — Association between DCC IHC status and T factors. [file 13148_2015_96_MOESM2_ESM.pptx]

## Slide 1
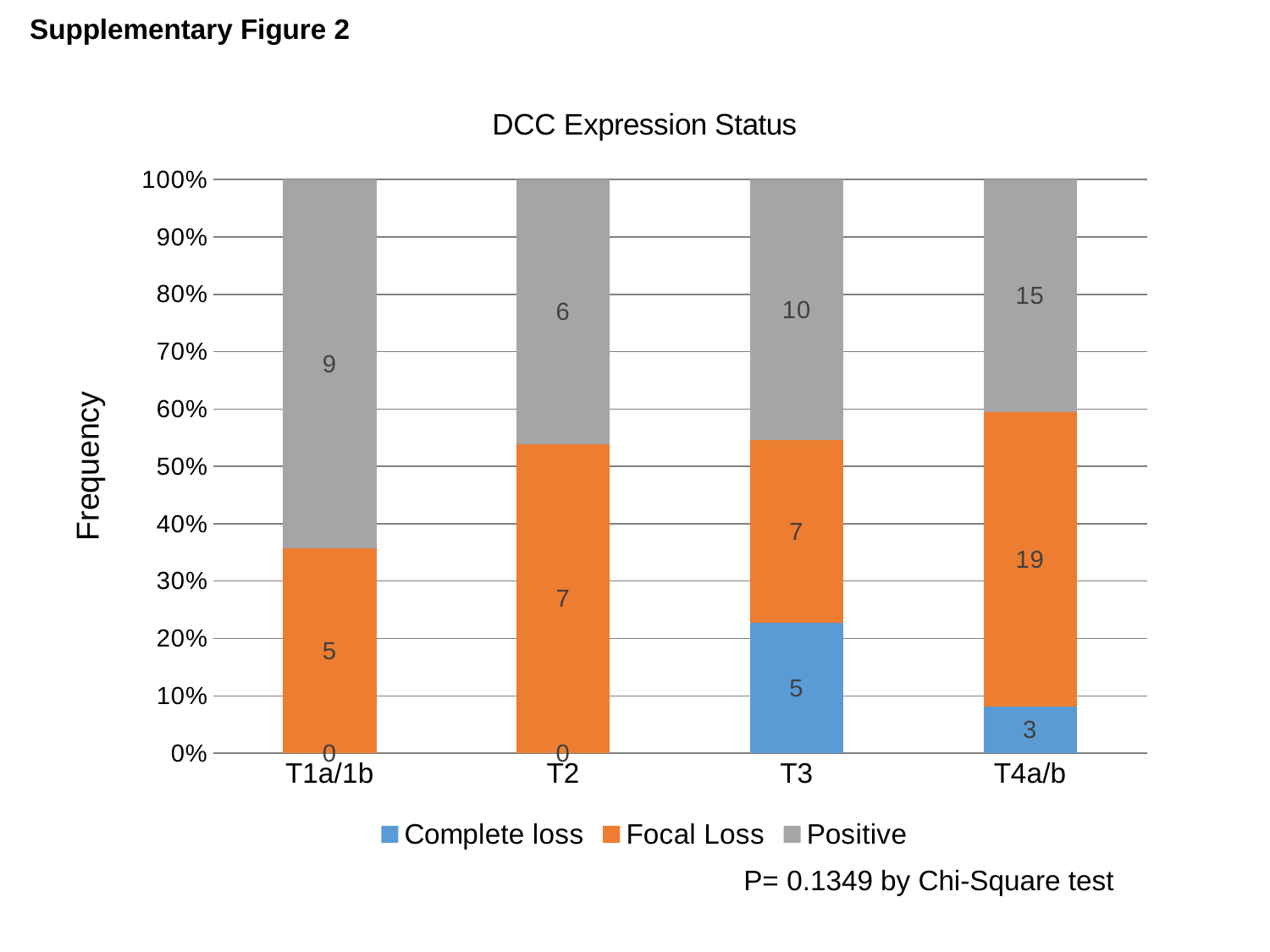

Supplementary Figure 2
### Chart: DCC Expression Status
| Category | Complete loss | Focal Loss | Positive |
|---|---|---|---|
| T1a/1b | 0.0 | 5.0 | 9.0 |
| T2 | 0.0 | 7.0 | 6.0 |
| T3 | 5.0 | 7.0 | 10.0 |
| T4a/b | 3.0 | 19.0 | 15.0 |Frequency
P= 0.1349 by Chi-Square test
