# Supplementary material for: Genetic and epigenetic alterations of netrin-1 receptors in gastric cancer with chromosomal instability
Source: Clin Epigenetics. 2015 Jul 23;7(1):73. doi: 10.1186/s13148-015-0096-y (PMC4511994; doi:10.1186/s13148-015-0096-y)
Supplement: Additional file 3: Figure S3. — Splicing variants of UNC5C mRNA and location of the primers which can distinguish expression status of the splicing variants. [file 13148_2015_96_MOESM3_ESM.pptx]

## Slide 1
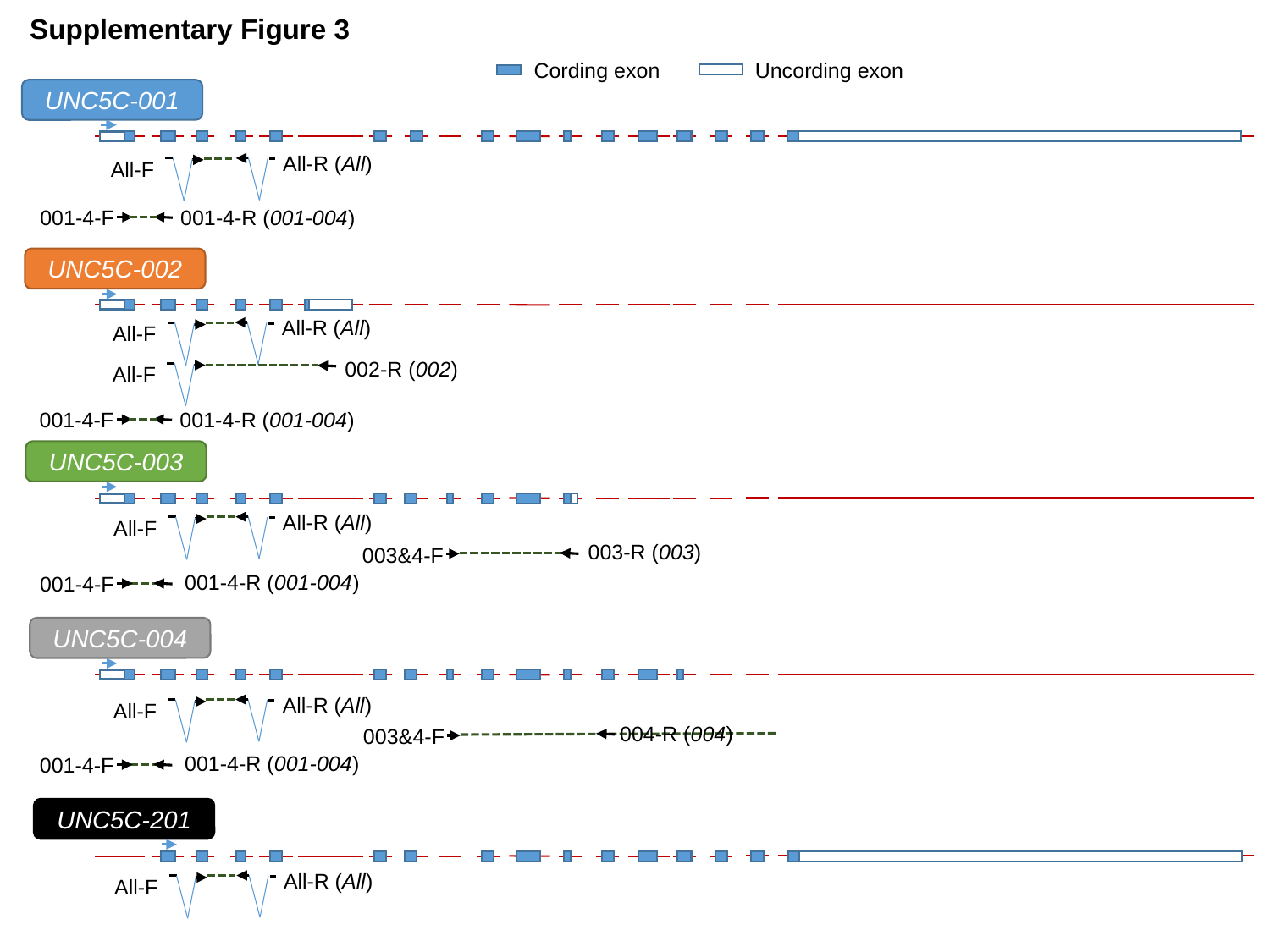

Supplementary Figure 3
Uncording exon
Cording exon
UNC5C-001
All-R (All)
All-F
001-4-F
001-4-R (001-004)
UNC5C-002
All-R (All)
All-F
002-R (002)
All-F
001-4-F
001-4-R (001-004)
UNC5C-003
All-R (All)
All-F
003-R (003)
003&4-F
001-4-R (001-004)
001-4-F
UNC5C-004
All-R (All)
All-F
004-R (004)
003&4-F
001-4-R (001-004)
001-4-F
UNC5C-201
All-R (All)
All-F
