# Supplementary material for: Genetic and epigenetic alterations of netrin-1 receptors in gastric cancer with chromosomal instability
Source: Clin Epigenetics. 2015 Jul 23;7(1):73. doi: 10.1186/s13148-015-0096-y (PMC4511994; doi:10.1186/s13148-015-0096-y)
Supplement: Additional file 4: Figure S4. — Expression status of splicing variants of UNC5C mRNA and association of UNC5C methylation for 10 gastric cancer cell lines. Ten gastric cancer cell lines and NHLF cells were analyzed for mRNA expression by RT-PCR of UNC5C splicing variants and beta-actin genes. The lowest panel illustrates the methylation profile obtained from COBRA. [file 13148_2015_96_MOESM4_ESM.pptx]

## Slide 1
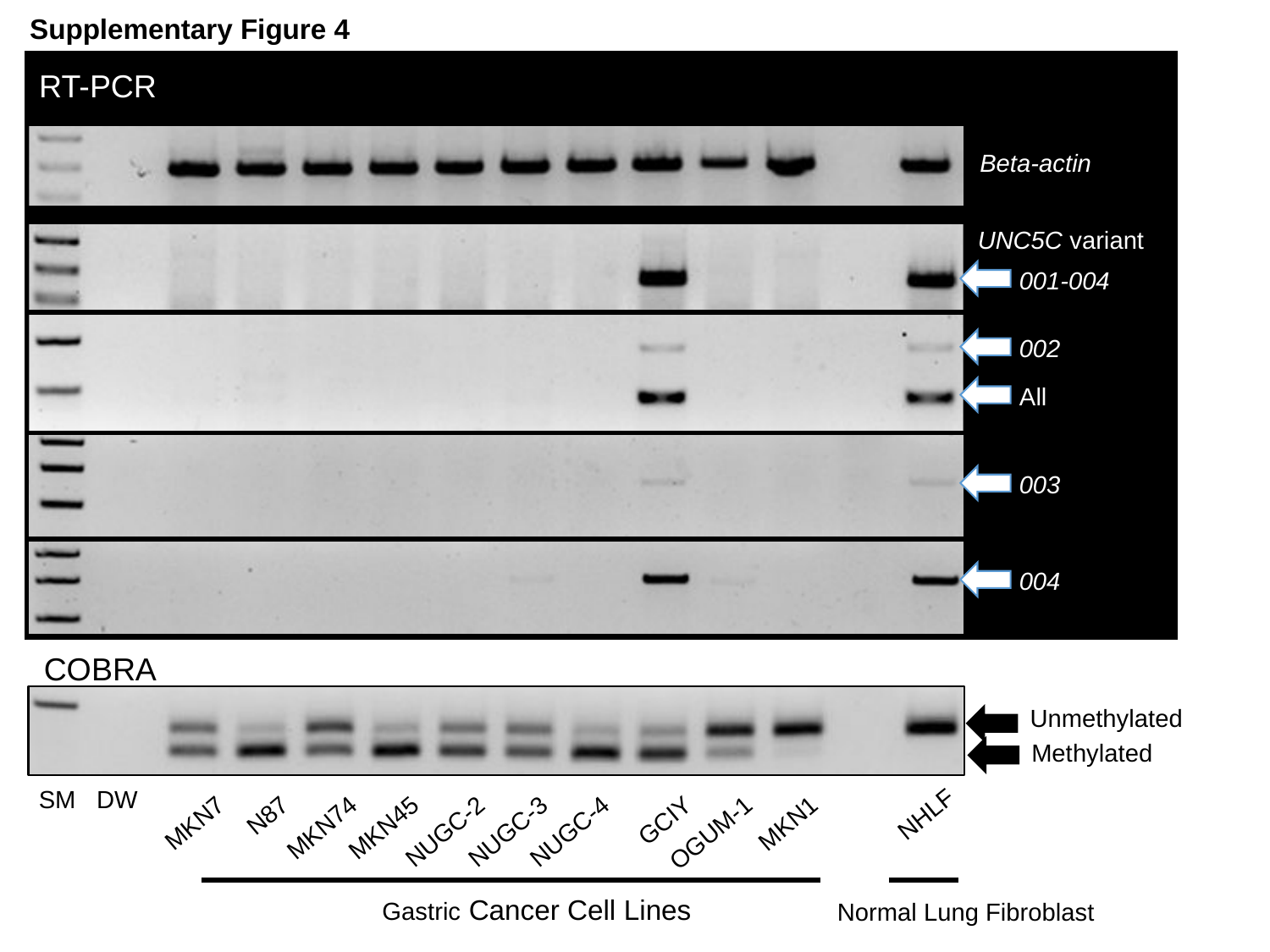

Supplementary Figure 4
RT-PCR
Beta-actin
UNC5C variant
001-004
002
All
003
004
COBRA
Unmethylated
Methylated
NHLF
N87
GCIY
MKN7
MKN1
SM
DW
MKN74
MKN45
NUGC-2
NUGC-3
NUGC-4
OGUM-1
Gastric Cancer Cell Lines
Normal Lung Fibroblast
