# Supplementary material for: Genetic and epigenetic alterations of netrin-1 receptors in gastric cancer with chromosomal instability
Source: Clin Epigenetics. 2015 Jul 23;7(1):73. doi: 10.1186/s13148-015-0096-y (PMC4511994; doi:10.1186/s13148-015-0096-y)
Supplement: Additional file 5: Figure S5. — Association of UNC5C methylation and loss of UNC5C mRNA expression in gastric cancer and colorectal cancer cell lines. Two gastric cancer cell lines, three colorectal cancer cell lines, and CCD18Co cells were analyzed for mRNA expression by RT-PCR of UNC5C and beta-actin genes. The lowest panel illustrates the methylation profile obtained from COBRA. [file 13148_2015_96_MOESM5_ESM.pptx]

## Slide 1
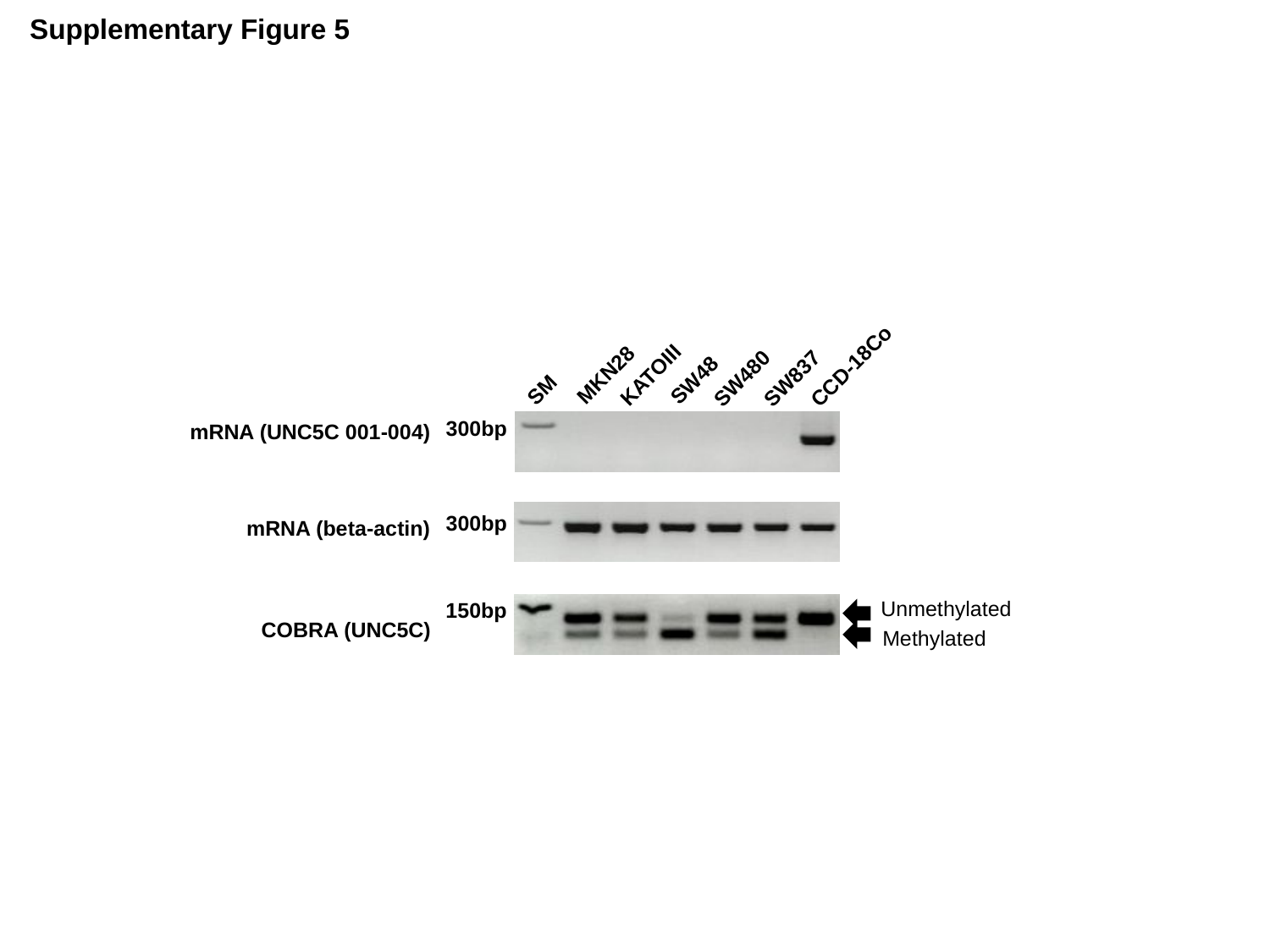

Supplementary Figure 5
CCD-18Co
MKN28
KATOIII
SW480
SW837
SW48
SM
300bp
mRNA (UNC5C 001-004)
300bp
mRNA (beta-actin)
150bp
COBRA (UNC5C)
Unmethylated
Methylated
