# Supplementary material for: Genetic and epigenetic alterations of netrin-1 receptors in gastric cancer with chromosomal instability
Source: Clin Epigenetics. 2015 Jul 23;7(1):73. doi: 10.1186/s13148-015-0096-y (PMC4511994; doi:10.1186/s13148-015-0096-y)
Supplement: Additional file 7: Figure S6. — Association between alteration patterns in netrin-1 receptors and clinicopathological features in gastric cancers. Correlation between alterations in netrin-1 receptors and gender (A), histology (B), H. pylori cagA expression status (C), and (D) age. In the box plot diagrams, the horizontal line within each box represents the median, the limits of each box are the interquartile ranges, and the whiskers denote the maximum and minimum values. Asterisks plus numbers denote mean age at surgery. [file 13148_2015_96_MOESM7_ESM.pptx]

## Slide 1
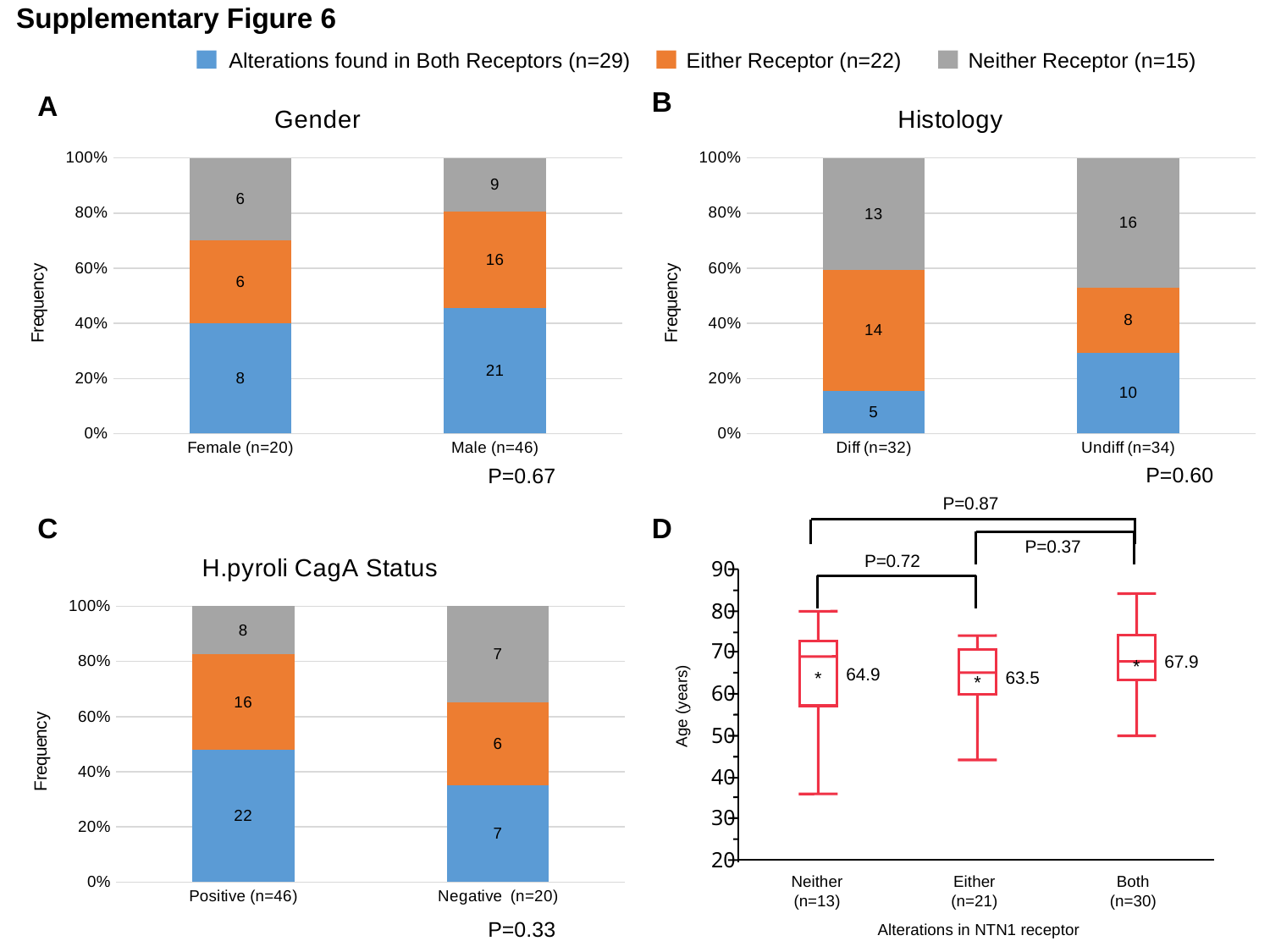

Supplementary Figure 6
Alterations found in Both Receptors (n=29)
Either Receptor (n=22)
Neither Receptor (n=15)
B
A
### Chart: Gender
| Category | Both Receptors | Either | Neither |
|---|---|---|---|
| Female (n=20) | 8.0 | 6.0 | 6.0 |
| Male (n=46) | 21.0 | 16.0 | 9.0 |
### Chart: Histology
| Category | Both Receptors | Either | Neither |
|---|---|---|---|
| Diff (n=32) | 5.0 | 14.0 | 13.0 |
| Undiff (n=34) | 10.0 | 8.0 | 16.0 |P=0.60
P=0.67
P=0.87
P=0.37
P=0.72
90
*
80
*
*
70
67.9
64.9
63.5
60
Age (years)
50
40
30
20
Neither
(n=13)
Either
(n=21)
Both
(n=30)
Alterations in NTN1 receptor
C
D
### Chart: H.pyroli CagA Status
| Category | Both Receptors | Either | Neither |
|---|---|---|---|
| Positive (n=46) | 22.0 | 16.0 | 8.0 |
| Negative (n=20) | 7.0 | 6.0 | 7.0 |P=0.33
